# Frailty and Early Extubation Independently Influence Postoperative Recovery Following Liver Transplantation—A Retrospective Cohort Study

**DOI:** 10.1155/joot/5545323

**Published:** 2026-04-28

**Authors:** Ishan Antony, Noor Albusta, Zhibang Lin, Ryan Nazemian, Bartholomew J. Kane, Ming Valerie Lin

**Affiliations:** ^1^ Department of Internal Medicine, Lahey Hospital & Medical Center, Burlington, Massachusetts, USA, lahey.org; ^2^ Comparative Effectiveness Research Institute, Lahey Hospital and Medical Center, Burlington, Massachusetts, USA; ^3^ Department of Anesthesiology, Perioperative and Pain Medicine, Lahey Hospital & Medical Center, Burlington, Massachusetts, USA, lahey.org; ^4^ Department of Surgery, Division of Hepatobiliary and Transplant Surgery, Lahey Hospital & Medical Center, Burlington, Massachusetts, USA, lahey.org

**Keywords:** early extubation, frailty, length of stay, liver transplantation, recovery

## Abstract

**Background:**

Frailty and perioperative management critically influence outcomes following liver transplantation (LT). The liver frailty index (LFI) objectively assesses frailty, while early extubation (EE) has been linked to enhanced recovery in surgical patients. However, the associations of frailty and EE with postoperative outcomes in LT remain unclear.

**Methods:**

This retrospective cohort study included adult LT recipients at a tertiary care center between 1/2019 and 7/2023. Patients were classified as frail (LFI ≥ 4.5) or nonfrail (LFI < 4.5) and stratified by EE versus delayed extubation (DE). Primary outcomes were EE rate, ICU length of stay (LOS), and hospital LOS. Linear regression models adjusted for age, gender, BMI, MELD, transplant type, and liver disease etiology.

**Results:**

Of 158 postliver transplant patients, 38 (24.1%) were frail. Frail patients had longer ICU LOS (33.2 vs. 4.2 days, *p* < 0.001) and hospital LOS (59.5 vs. 10.1 days, *p* < 0.001) compared to nonfrail patients. EE occurred in 78.5% of all postliver transplant patients, with no significant difference by frailty (*p* = 0.821). Multivariable regression showed frailty was associated with increased hospital LOS (*β* = +48 days, 95% CI: 43–54, *p* < 0.001) and ICU LOS (*β* = +28 days, 95% CI: 25–32, *p* < 0.001), while EE was associated with decreased hospital LOS (*β* = −12 days, 95% CI: −17 to −6.7, *p* < 0.001) and ICU LOS (*β* = −12 days, 95% CI: −15 to −8.4, *p* < 0.001), irrespective of frailty status.

**Conclusions:**

Our study demonstrated that EE significantly shortens ICU and hospital stays in postliver transplant patients with pretransplant frailty. These findings underscore the importance of incorporating EE protocols in this high‐risk population and warrant further investigation into strategies that facilitate implementation to optimize clinical outcomes.

**Social media post**
 Frailty prolongs recovery after liver transplant, but EE reduces hospital stay regardless of frailty. Frailty assessment + EE = optimized post‐LT recovery. #LiverTransplant

## 1. Introduction

Frailty is defined as a clinical state of decreased physiological reserves and increased vulnerability to health stressors. Frailty is increasingly being recognized as a critical determinant of outcomes in liver transplantation (LT), influencing recovery, hospital resource utilization, and survival [1]. Traditional frailty assessments often rely on subjective methods, which can introduce variability and limit clinical utility. Of the many frailty measurement tools available, the liver frailty index has wider applicability in the ambulatory scenario, given its low cost, ease of use, interrater reliability, and repeatability. The liver frailty index (LFI) is a tool designed to measure physical frailty in patients with cirrhosis and aims to identify patients at higher risk of mortality and poor outcomes while on the liver transplant (LT) waitlist. LFI includes measures of grip strength, chair stand, and balance testing. The LFI is a continuous metric, where a higher number indicates a greater degree of physical functional impairment. LFI offers an objective and validated measure of frailty, providing a consistent approach for risk stratification in LT candidates [1].

Early extubation (EE), typically defined as intraoperative or immediate postoperative removal of the endotracheal tube, has been linked to enhanced recovery after LT by reducing ventilator‐associated complications, facilitating earlier mobilization, and shortening ICU stays. By minimizing the physical and psychological burden of prolonged intubation, EE can promote faster recovery. Furthermore, EE has been shown to lower the incidence of postoperative complications such as respiratory infections and delirium, which are particularly detrimental in patients with limited physiological reserves [2]. While EE has demonstrated benefits in overall LT populations [2, 3], its feasibility and impact among frail postliver transplant patients with limited physiological reserves have not been well characterized.

Understanding whether EE mitigates the adverse recovery trajectories associated with frailty could inform perioperative decision‐making and optimize post‐LT management. This study aimed to evaluate the association between frailty and the rate of EE and to assess their effects on ICU and hospital length of stay (LOS) in adult LT recipients. We hypothesize that implementing EE in frail liver transplant recipients could reduce the hospital and ICU LOS and improve overall clinical outcomes.

## 2. Methods

This is a single‐center retrospective cohort study which was approved by the Lahey Hospital and Medical Center institutional review board, and all data were de‐identified to protect patient confidentiality.

All adult patients (≥ 18 years) who underwent LT at our center between January 2019 and July 2023 were screened. Patients were excluded if they underwent multiorgan transplantation, had incomplete LFI or extubation data, or demised intraoperatively.

Frailty was assessed preoperatively using LFI as part of the routine pretransplant evaluation. Based on the established guidelines, patients were classified as frail (LFI ≥ 4.5) or nonfrail (LFI < 4.5). EE was defined as the removal of the endotracheal tube in the operating room (OR), while extubation outside of the OR was classified as delayed extubation (DE). By default, patients were considered for EE per institutional protocol; decisions for DE were based on intraoperative or immediate postoperative clinician judgment.

Data were collected retrospectively from electronic medical records which included demographic characteristics (age, sex, ethnicity, and BMI), clinical parameters (MELD score, liver disease etiology, and albumin level), and LFI and its subcomponent scores. The primary outcomes were EE rates, ICU LOS, and total hospital LOS. EE rates by frailty status were also assessed. Secondary outcomes included the association between frailty and liver disease etiology as well as the impact of demographic and clinical factors—including age, gender, BMI, ethnicity, and transplant type—on ICU/hospital stay and extubation status. Continuous variables are summarized as means with standard deviations (SD) or medians with interquartile ranges (IQR), depending on the data distribution. Categorical variables are presented as frequencies and percentages. Group comparisons were performed using independent *t*‐tests for normally distributed continuous variables, Mann–Whitney *U* tests for nonnormally distributed variables, and chi‐square (*χ* ^2^) tests for categorical data. Multivariable linear regression models were constructed to evaluate the independent associations of frailty and EE with ICU and hospital LOS, adjusting for age, gender, BMI, MELD score, transplant type, and liver disease etiology. The regression model formula used was: LOS = β0 + β1∗(EE) + β2∗(Frailty) + β3∗(Age) + β4∗(Gender) + β5∗(BMI) + β6∗(MELD) + β7∗(Transplant Type) + β8∗(Liver Etiology) + ε.

“LOS” = *s* for categorica∗(“Frailty”) + variable linear regression models were constructed to evaluate the independent associations of frailty and E. where EE = early extubation (yes = 1, no = 0), Frailty = LFI ≥ 4.5 (yes = 1, no = 0), Gender (male = 1, female = 0), Transplant Type (LDLT = 1, DDLT = 0), and *ε* = error term. Statistical significance was defined as *p* < 0.05, and analyses were performed using SPSS Version 30.0.

## 3. Results

### 3.1. Baseline Characteristics

A total of 158 adult liver transplant (LT) recipients with documented LFI scores were included in the analysis (Table 1).

**TABLE 1:** Baseline variables comparison between frailty and nonfrailty group.

	Nonfrail (*N* = 120)	Frail (*N* = 38)	Total (*N* = 158)	*p* value
*Age*				0.082^1^
Mean and SD	55.70 (10.68)	59.13 (10.10)	56.53 (10.61)	
Median	58.00	63.00	59.00	
Q1–Q3	49.50, 64.00	56.00, 65.00	51.00, 64.00	
Min	20.00	22.00	20.00	
Max	74.00	71.00	74.00	
*Gender*				0.156^2^
F	32 (26.7%)	15 (39.5%)	47 (29.7%)	
M	88 (73.3%)	23 (60.5%)	111 (70.3%)	
*Ethnicity*				0.007^2^
Asian, non‐Hispanic	5 (4.2%)	0 (0.0%)	5 (3.2%)	
Black, non‐Hispanic	1 (0.8%)	0 (0.0%)	1 (0.6%)	
Hispanic/Latino	2 (1.7%)	5 (13.2%)	7 (4.4%)	
Multiracial, non‐Hispanic	1 (0.8%)	2 (5.3%)	3 (1.9%)	
White, non‐Hispanic	111 (92.5%)	31 (81.6%)	142 (89.9%)	
*BMI*				0.112^1^
Mean and SD	28.82 (5.36)	30.38 (4.78)	29.20 (5.25)	
Median	27.85	30.50	28.69	
Q1–Q3	25.09, 32.08	27.54, 34.50	25.53, 32.89	
Min	17.67	19.27	17.67	
Max	43.72	38.37	43.72	
*Listed MELD*				0.117^1^
Mean and SD	22.35 (7.76)	24.63 (7.80)	22.90 (7.80)	
Median	22.50	25.50	23.00	
Q1–Q3	17.00, 28.00	18.00, 31.00	17.00, 29.00	
Min	6.00	10.00	6.00	
Max	40.00	40.00	40.00	
*Natural MELD-NA on DOS*				0.062^1^
Mean and SD	20.76 (8.64)	23.68 (7.44)	21.46 (8.44)	
Median	20.00	24.50	21.00	
Q1–Q3	15.00, 28.00	17.25, 29.75	15.00, 29.00	
Min	6.00	10.00	6.00	
Max	41.00	38.00	41.00	
*Transplant type*				0.046^2^
DDLT	88 (73.3%)	34 (89.5%)	122 (77.2%)	
LDLT	32 (26.7%)	4 (10.5%)	36 (22.8%)	
*Liver etiology*				0.583^2^
Alcoholic	60 (50.0%)	21 (55.3%)	81 (51.3%)	
Nonalcoholic	60 (50.0%)	17 (44.7%)	77 (48.7%)	

^1^Linear model ANOVA.

^2^Fisher’s exact test for count data.

Of these, 38 (24.1%) were frail (LFI ≥ 4.5) and 120 (75.9%) were nonfrail (LFI < 4.5). The mean age was 56.5 ± 10.6 years, with frail patients being older (59.1 ± 10.1 vs. 55.7 ± 10.7 years, *p* = 0.082). Males comprised 70.3% of the cohort, with no significant gender differences by frailty (*p* = 0.156). The mean BMI was 29.2 ± 5.3, with frail patients trending higher (30.4 ± 4.8 vs. 28.8 ± 5.4, *p* = 0.112). The mean listed MELD score was 22.9 ± 7.8, with no significant difference by frailty (*p* = 0.117). The liver disease etiology of the study population showed 51.3% (81/158) had alcoholic liver disease and 48.7% (77/158) had nonalcoholic liver disease. Among the frail patients, 55.3% (21/38) had alcoholic liver disease, and 44.7% (17/38) had nonalcoholic liver disease, compared to 50% (60/120) and 50% (60/120), respectively, in the nonfrail patients. There was no statistically significant association between alcoholic liver disease and nonalcoholic liver disease and frailty status (*p* = 0.583).

Deceased donor liver transplantation (DDLT) was more common in frail patients (89.5%) compared to nonfrail patients (73.3%, *p* = 0.046). Hispanic/Latino (13.2% frail) and multiracial non‐Hispanic (individuals who identify with two or more racial groups but do not identify as Hispanic or Latino) (5.3% frail) patients had higher proportions of frailty compared to Asian and Black non‐Hispanic patients, who had no frail individuals (*p* = 0.007). Frail patients had significantly lower handgrip strength across all measures (*p* < 0.001) with average handgrip strength of 19.6 kg (SD 7.7) in frail vs. 30.1 kg (SD 8.8) in nonfrail. Chair stand times were longer in frail patients but did not reach statistical significance when compared to the nonfrail patients (14.5 ± 12.3 vs. 11.9 ± 5.0 s, *p* = 0.056) (Table 2).

**TABLE 2:** Targeted outcome variables comparison between frailty and nonfrailty group.

	Nonfrail (*N* = 120)	Frail (*N* = 38)	Total (*N* = 158)	*p* value
*PT: Hand grip 1*				< 0.001^1^
Mean and SD	30.06 (8.98)	19.49 (7.33)	27.52 (9.71)	
Median	29.10	18.60	26.75	
Q1–Q3	23.45, 35.58	14.80, 23.60	20.50, 34.88	
Min	11.80	2.70	2.70	
Max	62.60	39.50	62.60	
*PT: Hand grip 2*				< 0.001^1^
Mean and SD	30.31 (8.98)	19.34 (7.72)	27.67 (9.87)	
Median	30.15	18.95	27.00	
Q1–Q3	23.37, 37.25	15.53, 24.50	20.70, 34.18	
Min	12.80	2.50	2.50	
Max	62.60	37.40	62.60	
*PT: Hand grip 3*				< 0.001^1^
Mean and SD	29.84 (9.22)	20.00 (8.26)	27.48 (9.92)	
Median	29.65	19.90	27.15	
Q1–Q3	23.75, 36.23	14.62, 26.15	20.38, 34.48	
Min	0.80	2.40	0.80	
Max	61.20	41.00	61.20	
*PT: Hand grip average*				< 0.001^1^
Mean and SD	30.07 (8.82)	19.61 (7.68)	27.56 (9.64)	
Median	29.52	18.93	26.88	
Q1–Q3	23.59, 36.03	14.64, 25.04	20.33, 34.57	
Min	12.80	2.53	2.53	
Max	62.13	38.93	62.13	
*PT: 5 chair stands*				0.056^1^
Mean and SD	11.88 (4.97)	14.53 (12.25)	12.51 (7.44)	
Median	11.39	15.59	11.80	
Q1–Q3	9.14, 13.12	0.00, 19.62	9.05, 14.34	
Min	0.00	0.00	0.00	
Max	40.05	43.70	43.70	
*PT: Tandem walk*				< 0.001^1^
Mean and SD	9.60 (1.44)	5.98 (4.46)	8.73 (2.94)	
Median	10.00	9.00	10.00	
Q1–Q3	10.00, 10.00	0.00, 10.00	10.00, 10.00	
Min	0.00	0.00	0.00	
Max	10.00	10.00	10.00	
*Hospital length of stay*				< 0.001^1^
Mean and SD	10.07 (3.88)	59.53 (29.78)	21.97 (25.88)	
Median	9.00	63.00	11.00	
Q1–Q3	7.00, 12.00	33.75, 73.75	8.00, 19.00	
Min	3.00	24.00	3.00	
Max	20.00	154.00	154.00	
*ICU length of stay*				< 0.001^1^
Mean and SD	4.22 (3.22)	33.18 (19.91)	11.19 (15.98)	
Median	3.00	28.00	5.00	
Q1–Q3	2.00, 6.25	19.50, 40.50	2.00, 12.00	
Min	1.00	12.00	1.00	
Max	13.00	102.00	102.00	
*Albumin level*				0.084^1^
Mean and SD	2.99 (0.65)	2.78 (0.62)	2.94 (0.65)	
Median	3.00	2.60	2.90	
Q1–Q3	2.50, 3.40	2.40, 3.20	2.42, 3.30	
Min	1.10	1.20	1.10	
Max	4.70	4.40	4.70	
*Extubation in OR*				0.821^2^
N	25 (20.8%)	9 (23.7%)	34 (21.5%)	
Y	95 (79.2%)	29 (76.3%)	124 (78.5%)	

^1^Linear model ANOVA.

^2^Fisher’s exact test for count data.

### 3.2. EE Rates

EE was achieved in 124 (78.5%) patients across the entire cohort (Table 3). When stratified by frailty status, EE rates were not significantly different between frail and nonfrail patients (76.3% vs. 79.2%, *p* = 0.821) (Table 2). Patients who underwent EE were significantly younger than those with DE (55.3 ± 9.8 vs. 60.0 ± 9.3 years, *p* = 0.014). MELD scores were lower in the EE group compared to the DE group (21.7 ± 6.5 vs. 25.6 ± 7.8, *p* = 0.003). Serum albumin levels were higher in patients who underwent EE (3.0 ± 0.5 vs. 2.7 ± 0.4 g/dL, *p* = 0.003). Patients who underwent EE demonstrated significantly higher handgrip strength compared to those with DE (30.1 ± 9.1 kg vs. 24.9 ± 8.2 kg, *p* = 0.002). While EE patients completed a higher number of chair stands (mean 3.8 ± 2.4 vs. 3.3 ± 2.3), this difference was not statistically significant (*p* = 0.243). Balance times were similar between the two groups (14.1 ± 2.2 vs. 14.2 ± 1.8 s, *p* = 0.805), suggesting no measurable difference in balance performance by extubation status (Table 3). There were no significant differences in gender, BMI, ethnicity, or transplant type between EE and DE groups (Table 4).

**TABLE 3:** Targeted outcome variables comparison between EE and non‐EE group.

	*Y* (*N* = 124)	*N* (*N* = 34)	Total (*N* = 158)	*p* value
*PT: Hand grip 1*				0.172^1^
Mean and SD	28.07 (10.13)	25.50 (7.79)	27.52 (9.71)	
Median	27.80	23.95	26.75	
Q1–Q3	20.70, 35.25	20.52, 29.98	20.50, 34.88	
Min	2.70	14.80	2.70	
Max	62.60	44.60	62.60	
*PT: Hand grip 2*				0.230^1^
Mean and SD	28.17 (10.26)	25.87 (8.15)	27.67 (9.87)	
Median	27.30	24.95	27.00	
Q1–Q3	19.90, 35.85	20.97, 31.10	20.70, 34.18	
Min	2.50	10.70	2.50	
Max	62.60	45.00	62.60	
*PT: Hand grip 3*				0.370^1^
Mean and SD	27.85 (10.40)	26.12 (7.87)	27.48 (9.92)	
Median	27.50	26.05	27.15	
Q1–Q3	20.23, 35.02	20.68, 31.60	20.38, 34.48	
Min	0.80	12.50	0.80	
Max	61.20	46.40	61.20	
*PT: Hand grip average*				0.240^1^
Mean and SD	28.03 (10.06)	25.83 (7.82)	27.56 (9.64)	
Median	27.57	25.03	26.88	
Q1–Q3	20.00, 35.02	21.29, 29.52	20.33, 34.57	
Min	2.53	12.67	2.53	
Max	62.13	45.33	62.13	
*PT: 5 chair stands*				0.179^1^
Mean and SD	12.10 (6.56)	14.04 (10.00)	12.51 (7.44)	
Median	11.52	12.84	11.80	
Q1–Q3	9.01, 14.08	9.89, 14.72	9.05, 14.34	
Min	0.00	0.00	0.00	
Max	43.70	40.05	43.70	
*PT: Tandem walk*				0.254^1^
Mean and SD	8.87 (2.78)	8.22 (3.47)	8.73 (2.94)	
Median	10.00	10.00	10.00	
Q1–Q3	10.00, 10.00	8.88, 10.00	10.00, 10.00	
Min	0.00	0.00	0.00	
Max	10.00	10.00	10.00	
*Hospital length of stay*				0.005^1^
Mean and SD	18.97 (22.80)	32.91 (33.06)	21.97 (25.88)	
Median	9.00	16.50	11.00	
Q1–Q3	7.00, 17.00	13.00, 52.25	8.00, 19.00	
Min	3.00	10.00	3.00	
Max	154.00	125.00	154.00	
*ICU length of stay*				< 0.001^1^
Mean and SD	8.51 (11.98)	20.97 (23.54)	11.19 (15.98)	
Median	3.00	10.50	5.00	
Q1–Q3	2.00, 7.00	8.00, 28.00	2.00, 12.00	
Min	1.00	4.00	1.00	
Max	66.00	102.00	102.00	
*Albumin level*				0.067^1^
Mean and SD	2.99 (0.67)	2.76 (0.55)	2.94 (0.65)	
Median	2.95	2.85	2.90	
Q1–Q3	2.50, 3.40	2.30, 3.18	2.42, 3.30	
Min	1.10	1.50	1.10	
Max	4.70	3.80	4.70	

^1^Linear model ANOVA.

**TABLE 4:** Baseline variables comparison between EE and non‐EE group.

	*Y* (*N* = 124)	*N* (*N* = 34)	Total (*N* = 158)	*p* value
*Age*				0.402^1^
Mean and SD	56.15 (10.93)	57.88 (9.39)	56.53 (10.61)	
Median	59.00	60.00	59.00	
Q1–Q3	50.75, 64.00	53.00, 65.75	51.00, 64.00	
Min	20.00	35.00	20.00	
Max	74.00	72.00	74.00	
*Gender*				0.833^2^
F	36 (29.0%)	11 (32.4%)	47 (29.7%)	
M	88 (71.0%)	23 (67.6%)	111 (70.3%)	
*Ethnicity*				0.946^2^
Asian, non‐Hispanic	4 (3.2%)	1 (2.9%)	5 (3.2%)	
Black, non‐Hispanic	1 (0.8%)	0 (0.0%)	1 (0.6%)	
Hispanic/Latino	6 (4.8%)	1 (2.9%)	7 (4.4%)	
Multiracial, non‐Hispanic	2 (1.6%)	1 (2.9%)	3 (1.9%)	
White, non‐Hispanic	111 (89.5%)	31 (91.2%)	142 (89.9%)	
*BMI*				0.995^1^
Mean and SD	29.20 (5.24)	29.20 (5.38)	29.20 (5.25)	
Median	28.84	27.85	28.69	
Q1–Q3	25.43, 32.43	25.71, 33.73	25.53, 32.89	
Min	17.67	20.80	17.67	
Max	43.72	41.67	43.72	
*Listed MELD*				0.048^1^
Mean and SD	22.26 (7.60)	25.24 (8.20)	22.90 (7.80)	
Median	22.50	26.00	23.00	
Q1–Q3	17.00, 28.00	20.00, 31.50	17.00, 29.00	
Min	7.00	6.00	6.00	
Max	40.00	38.00	40.00	
*Natural MELD-NA on DOS*				0.021^1^
Mean and SD	20.65 (8.22)	24.41 (8.70)	21.46 (8.44)	
Median	20.00	25.00	21.00	
Q1–Q3	15.00, 27.00	18.25, 30.00	15.00, 29.00	
Min	6.00	6.00	6.00	
Max	41.00	38.00	41.00	
*Transplant Type*				0.821^2^
DDLT	95 (76.6%)	27 (79.4%)	122 (77.2%)	
LDLT	29 (23.4%)	7 (20.6%)	36 (22.8%)	
*Alcoholic*				0.439^2^
Alcoholic	66 (53.2%)	15 (44.1%)	81 (51.3%)	
Nonalcoholic	58 (46.8%)	19 (55.9%)	77 (48.7%)	

^1^Linear model ANOVA.

^2^Fisher’s exact test for count data.

### 3.3. Impact of Frailty and EE on LOS

Hospital LOS was significantly longer in frail patients (mean 59.5 days, SD 29.8) compared to nonfrail patients (mean 10.1 days, SD 3.9; *p* < 0.001). ICU LOS was also significantly longer in frail patients (mean 33.2 days, SD 19.9) compared to nonfrail patients (mean 4.2 days, SD 3.2; *p* < 0.001) (Table 2). The mean ICU LOS was significantly lower in the EE group (8.51 ± 11.98 days) than in the DE group (20.97 ± 23.54 days, *p* < 0.001). Similarly, the hospital LOS was shorter in EE patients (18.97 ± 22.80 days) compared to those in the DE group (32.91 ± 33.06 days, *p* = 0.005) (Table 3).

### 3.4. Multivariable Regression Analysis

In multivariable linear regression adjusting for age, gender, BMI, MELD score, transplant type, and liver disease etiology, frailty was independently associated with increased hospital LOS (*β* = +48 days, 95% CI: 43–54, *p* < 0.001) and ICU LOS (*β* = +28 days, 95% CI: 25–32, *p* < 0.001) (Tables 3 and 4). EE was independently associated with decreased hospital LOS (*β* = −12 days, 95% CI: −17 to −6.7, *p* < 0.001) and ICU LOS (*β* = −12 days, 95% CI: −15 to −8.4, *p* < 0.001) (Tables 5 and 6). Other covariates including age, gender, BMI, MELD, transplant type, and liver disease etiology were not significant predictors in either model. Multicollinearity testing confirmed no significant interaction between frailty and EE, indicating that the presence or absence of frailty did not statistically influence the likelihood of undergoing EE, such that both variables acted as independent predictors in the regression model.

**TABLE 5:** Linear regression model for hospital length of stay.

Characteristic	Beta	95% CI	*p* value
*Extubation in OR*			
N	—	—	
Y	−12	−17, −6.7	< 0.001
LFI > 4.5 (*Y* = 1, *N* = 0)	48	43, 54	< 0.001
Age	0.05	−0.17, 0.26	0.7

*Gender*			
F	—	—	
M	−3.8	−8.7, 1.1	0.13
BMI	−0.32	−0.75, 0.12	0.2
Listed MELD	0.20	−0.13, 0.53	0.2

*Transplant Type*			
DDLT	—	—	
LDLT	1.3	−4.9, 7.4	0.7

*Liver etiology*			
Alcoholic	—	—	
Nonalcoholic	−4.3	−8.7, 0.13	0.057

Abbreviation: CI = confidence interval.

**TABLE 6:** Linear regression model for ICU length of stay.

Characteristic	Beta	95% CI	*p* value
*Extubation in OR*			
N	—	—	
Y	−12	−15, −8.4	< 0.001
LFI > 4.5 (*Y* = 1, *N* = 0)	28	25, 32	< 0.001
Age	−0.01	−0.15, 0.13	0.9

*Gender*			
F	—	—	
M	−2.6	−5.7, 0.59	0.11
BMI	−0.12	−0.39, 0.16	0.4
Listed MELD	−0.04	−0.25, 0.18	0.7

*Transplant Type*			
DDLT	—	—	
LDLT	−0.73	−4.7, 3.2	0.7

*Liver etiology*			
Alcoholic	—	—	
Nonalcoholic	−2.7	−5.6, 0.12	0.061

Abbreviation: CI = confidence interval.

To further characterize the clinical impact of frailty and EE on postoperative recovery, we modeled the predicted LOS using multivariable regression estimates. The formula used is LOS days = 26.4 + EE status∗(‐12) + LFI status∗(48). Patients who were both frail and DE had the longest expected hospital LOS at 74.4 days, while nonfrail patients who received EE had the shortest hospital LOS at 14.4 days. Among frail patients, those who underwent EE had an average reduction of 12 days (i.e., 16.1% improvement) in hospital LOS compared to the DE group (62.4 vs. 74.4 days). Importantly, nonfrail patients with DE had a predicted LOS of 26.4 days. Similarly, to assess the impact of frailty and EE on ICU resource utilization, we modeled the predicted ICU LOS using multivariable linear regression. The formula applied is ICU LOS (days) = 4.3 + EE status × (−11) + LFI status × (24). Patients who were both frail and underwent DE had the longest predicted ICU LOS at 67.3 days, whereas nonfrail patients who received EE had the shortest ICU LOS at 6.7 days. Within the frail cohort, EE was associated with a reduction of 11 days, decreasing ICU LOS from 67.3 to 56.3 days, a 16.3% improvement. Nonfrail patients with DE had a predicted ICU LOS of 4.3 days.

## 4. Discussion

Prior studies have shown that frailty is associated with increased morbidity and healthcare utilization among LT candidates. Consistent with these observations, our findings demonstrate that frailty independently predicts prolonged ICU and hospital LOS following LT. However, our study demonstrated the implementation of EE in this patient population is associated with a significant reduction in both hospital and ICU LOS (up to 12 days) compared to DE. EE represents a practical, modifiable perioperative target that may attenuate frailty‐related adverse outcomes, thereby supporting the integration of EE protocols to enhance recovery.

EE after liver transplantation improves outcomes by reducing ventilator‐associated complications, optimizing hemodynamic stability, and promoting early mobilization. In contrast, prolonged mechanical ventilation increases risks of infection, respiratory compromise, and ICU‐acquired complications such as delirium, venous thromboembolism, and pressure ulcers [4]. EE limits sedation needs, lessens respiratory depression and delirium, and enables faster recovery of spontaneous breathing, oxygenation, and cardiovascular stability, which is crucial in post‐LT patients with fragile hemodynamics [5]. By facilitating early mobilization, EE shortens ICU and hospital stays, reduces deconditioning, and enhances functional recovery, thereby lowering the above‐mentioned risks. Collectively, these benefits support incorporating standardized EE protocols into perioperative liver transplant care [2].

Extubation practices vary widely among transplant centers, and some of the factors that contribute to DE include early postoperative pulmonary complications, hemodynamic instability, massive blood product transfusion, neurological status, a high MELD score, and the inherent nature of liver transplant surgery. A successful series of EE in LT has been reported since the 1990s [6]. A more recent study of 1555 patients at UCSF assessing the EE rates and trends over the past 10 years in liver transplant patients showed that 62% underwent EE in the OR, with only 3.2% of EE requiring reintubation [7]. A systematic review and meta‐analysis of short‐term outcomes after EE in LT showed lower ICU LOS, hospital LOS, and pulmonary complication rates in the early versus deferred extubation groups [8]. EE is an established part of fast‐track protocols in major surgeries, such as cardiac surgery; however, currently, there is no standard of care in liver transplantation. Our findings provide further support for EE as a key component of perioperative care, demonstrating its association with reduced ICU and hospital LOS even in a frail cohort.

In our study, EE did not differ significantly between frail and nonfrail patients, suggesting that frailty alone does not preclude successful EE when clinically appropriate. However, among frail patients, those who underwent EE had notably better outcomes than those who did not. Specifically, frail patients with EE had an estimated hospital LOS of 62.4 days, compared to 74.4 days for frail patients with DE, a reduction of 12 days, representing a 16.1% improvement. This is a clinically meaningful benefit in a high‐risk population and demonstrates that EE can tangibly reduce the burden of prolonged hospitalization, even in the context of frailty. ICU LOS showed similar trends, with a reduction from 28.3 days to 17.3 days in frail patients who underwent EE. While the nonfrail and EE group had the shortest LOS overall (14.4 hospital days and 4.3 ICU days), the comparison within the frail cohort reveals that EE still delivers substantial value in mitigating adverse outcomes. This aligns with prior studies showing that, with careful selection and intraoperative management, EE can be safely achieved in frail patients, potentially mitigating frailty‐related risks [9, 10]. Our findings challenge the notion that frail patients should be excluded from EE protocols and highlights that EE is a modifiable factor rather than a mere reflection of preoperative status. Early identification and targeted prehabilitation in frail LT candidates should be emphasized, as improving physical function before transplantation may further enhance postoperative recovery [11]. Additionally, standardized EE protocols tailored to frail patients could reduce mechanical ventilation time and postoperative complications.

Our study also noted an important finding that hand grip strength may be used as a surrogate marker for EE in this patient population, especially in patients who are immobilized due to various reasons during hospitalization.

This study has several limitations that should be considered when interpreting the findings. As a retrospective cohort study, it is subject to inherent biases, including selection bias and potential confounding variables, which may not have been fully accounted for. The study was conducted at a single tertiary care center, which may limit the generalizability of the results to other transplant centers with different perioperative management protocols, patient populations, or healthcare resources. Additionally, frailty assessments were available for only a subset of patients due to operational constraints. Variability in clinical decision‐making regarding EE may have influenced the results because the decision to extubate early was based on clinician judgment rather than a standardized protocol. This introduces the possibility of confounding by indication, whereby patients selected for EE may have had more favorable intraoperative physiology or clinical stability not fully captured by measured variables such as LFI or MELD score. Factors beyond frailty, such as intraoperative hemodynamics, blood loss, vasopressor requirements, graft quality, and surgical complexity, could influence both extubation timing and postoperative recovery. Furthermore, the study primarily focused on LOS as the primary outcome without evaluating long‐term survival, functional recovery, quality of life, or postoperative complications, which are critical for understanding the full impact of frailty and EE. Despite using multivariable analyses to adjust for key factors, unmeasured confounders such as nutritional status, sarcopenia severity, and social determinants of health may have influenced the findings. The absence of intraoperative hemodynamic, graft quality, and postoperative complication data limits the ability to fully attribute outcomes solely to frailty or extubation status. Future multicenter prospective studies incorporating standardized perioperative protocols and long‐term functional assessments are needed to validate these results and refine clinical decision‐making for frail LT recipients.

This study highlights the independent impact of frailty and EE on postoperative outcomes following LT. Frailty remains a strong predictor of prolonged ICU and hospital stays, while EE emerges as a modifiable factor that can enhance recovery across frailty strata. These findings support the integration of objective frailty assessments into LT risk stratification and the adoption of EE protocols to optimize patient outcomes and healthcare resource utilization in LT recipients.

## Funding

No funding was received for this manuscript.

## Disclosure

This research received no specific grant from any funding agency in the public, commercial, or not‐for‐profit sectors.

## Conflicts of Interest

The authors declare no conflicts of interest.

## Data Availability

The data that support the findings of this study are available from the corresponding author upon reasonable request.
